# Temporal dynamic in the impact of COVID− 19 outbreak on cause-specific mortality in Guangzhou, China

**DOI:** 10.1186/s12889-021-10771-3

**Published:** 2021-05-08

**Authors:** Li Li, Dong Hang, Han Dong, Chen Yuan-Yuan, Liang Bo-Heng, Yan Ze-Lin, Yang Zhou, Ou Chun-Quan, Qin Peng-Zhe

**Affiliations:** 1 State Key Laboratory of Organ Failure Research, Department of Biostatistics, Guangdong Provincial Key Laboratory of Tropical Disease Research, School of Public Health, Southern Medical University, Guangzhou, 510515 China; 2 Guangzhou Center for Disease Control and Prevention, Guangzhou, 510440 Guangdong China

**Keywords:** Coronavirus disease 2019, Excess mortality, Temporal dynamic, Sociodemographic status, Guangzhou, China

## Abstract

**Background:**

Studies related to the SARS-CoV-2 spikes in the past few months, while there are limited studies on the entire outbreak-suppressed cycle of COVID-19. We estimate the cause-specific excess mortality during the complete circle of COVID-19 outbreak in Guangzhou, China, stratified by sociodemographic status.

**Methods:**

Guangzhou Center for Disease Control Prevention provided the individual data of deaths in Guangzhou from 1 January 2018 through 30 June 2020. We applied Poisson regression models to daily cause-specific mortality between 1 January 2018 and 20 January 2020, accounting for effects of population size, calendar time, holiday, ambient temperature and PM_2.5_. Expected mortality was estimated for the period from 21 January through 30 June 2020 assuming that the effects of factors aforementioned remained the same as described in the models. Excess mortality was defined as the difference between the observed mortality and the expected mortality. Subgroup analyses were performed by place of death, age group, sex, marital status and occupation class.

**Results:**

From 21 January (the date on which the first COVID-19 case occurred in Guangzhou) through 30 June 2020, there were three stages of COVID-19: first wave, second wave, and recovery stage, starting on 21 January, 11 March, and 17 May 2020, respectively. Mortality deficits were seen from late February through early April and in most of the time in the recovery stage. Excesses in hypertension deaths occurred immediately after the starting weeks of the two waves. Overall, we estimated a deficit of 1051 (95% eCI: 580, 1558) in all-cause deaths. Particularly, comparing with the expected mortality in the absence of COVID-19 outbreak, the observed deaths from pneumonia and influenza substantially decreased by 49.2%, while deaths due to hypertension and myocardial infarction increased by 14.5 and 8.6%, respectively. In-hospital all-cause deaths dropped by 10.2%. There were discrepancies by age, marital status and occupation class in the excess mortality during the COVID-19 outbreak.

**Conclusions:**

The excess deaths during the COVID-19 outbreak varied by cause of death and changed temporally. Overall, there was a deficit in deaths during the study period. Our findings can inform preparedness measures in different stages of the outbreak.

**Supplementary Information:**

The online version contains supplementary material available at 10.1186/s12889-021-10771-3.

## Introduction

Coronavirus disease 2019 (COVID-19) pandemic is a serious threat for public health and the associated mortality burden may be comparable to the 1918 influenza pandemic which was estimated to cause at least 50 million deaths worldwide [1, 2]. On 11 March 2020, World Health Organization declared COVID-19 as a pandemic. Currently, COVID-19 continues spreading throughout the world. At the time of writing, there have been over 65.8 million confirmed infections and 1.5 million deaths of COVID-19 [3]. Understanding the impact of COVID-19 outbreak on mortality can inform resource allocation and help evaluate the effect of policy response to the outbreak. Excess mortality is a metric that has been commonly used to quantify the increased deaths compared with the expected mortality and the metric can indicate the death toll in a pandemic [4].

Excess mortality during the COVID-19 outbreak is very likely to change over time due to the evolution of SARS-CoV-2 virus and the fluctuation in other influencing factors of death (e.g. self-protection behavior). That is, the excess mortality should be distinct in different stages of the outbreak. The assessment of the temporal variation in the cause-specific excess mortality occurring in differential places (i.e. in hospitals vs outside hospitals) would shed light on the mechanisms on how the COVID-19 outbreak affected mortality and subsequently better direct care and interventions that aim to mitigate the negative effects of COVID-19 in the pandemic and post-pandemic periods. Currently, COVID-19 is spreading in many regions which are in the early or middle stages of the outbreak. Correspondingly, most of existing studies focused on the excess mortality in these stages [5–12]. To our knowledge, no prior study presents the excess mortality in the recovery stage of COVID-19. In addition, only a few studies or reports explored the cause-specific excess mortality during the COVID-19 outbreak [13–16]. More data are needed to further elucidate the process and the mechanisms on how the COVID-19 outbreak influenced mortality. Furthermore, examining the excess mortality by sociodemographic status could reveal the inequalities in the effects of COVID-19 outbreak and further illuminate targeted approaches to reducing the adverse effects of the outbreak. Age and gender disparities have been explored previously [6–9, 11–13, 17–20]. To our knowledge, there is no previous study estimating the excess deaths for subpopulations of other sociodemographic status, such as marital status and occupation class.

Excess mortality during the COVID-19 outbreak can vary across locations due to the disparities in the scale and intensity of the COVID-19, effectiveness of interventions, resilience of health and social care system and preparedness to the resurgence of COVID-19 [17]. Guangzhou is an international transportation hub with 15.3 million residents, locating in the south of China. On 21 January 2020, the first imported case of COVID-19 was reported in Guangzhou. A total of 557 symptomatic COVID-19 cases were reported from 21 January through 30 June 2020 [21]. Since 2 May 2020, there have been no local cases reported for more than 5 months. Guangzhou is one of the cities that have experienced the early, middle as well as recovery stages of the COVID-19 outbreak. Assessing the temporal trend in the mortality impact of the COVID-19 outbreak in Guangzhou would inform better preparedness for possible resurgence in the city in the future and give an indication of how to get ready for the COVID-19 outbreak for other cities which are now suffering from COVID-19, especially for those in the early and middle stages.

Guangzhou has a sophisticated death registration system. The data recorded in the system includes but not limited to cause of death, various sociodemographic variables and place of death. The abundant information of deaths allows for a comprehensive analysis of the mortality impact of COVID-19 outbreak. In this study, we estimate the excess mortality of COVID-19 outbreak from a spectrum of causes of death and for different subgroups, and delineate the temporal variation in the excess mortality of the complete circle of COVID-19 outbreak in Guangzhou, China from 21 January through 30 June 2020.

## Methods

### Data sources

Information on the number of COVID-19 cases was collected from the website of Guangzhou Health Commission. Guangzhou Health Commission started to report asymptomatic SARS-CoV-2 cases on 31 March 2020. We extracted the individual data of deaths occurring in Guangzhou between 1 January 2018 and 30 June 2020 from the death registration system operated by Guangzhou Center for Disease Control Prevention (CDC). The information of each death included date of birth, date of death, age, sex, marital status, occupation class, cause of death and place of death. The underlying causes of death were coded and classified using the International Statistical Classification of Diseases and Related Health Problems 10th Revision (ICD-10). In the current study, we considered six main categories of causes of death, including all causes (ICD-10: A00-Z99), respiratory diseases (J00-J99), cardiovascular diseases (I00-I99), malignant neoplasms (C00-C97), diabetes mellitus (E10-E14) and external causes (V00-Y99), and nine subcategories, including pneumonia and influenza (J09-J18), chronic lower respiratory diseases (J40-J47), hypertension (I10-I15), myocardial infraction (I21-I23), cerebrovascular diseases (I60-I69), malignant neoplasm of liver and intrahepatic bile dusts (C22), malignant neoplasms of the trachea, bronchus and lung (C33-C34), transport accidents (V01-V99) and intentional self-harm (X60-X84). Data on annual population size were obtained from Public Security Bureau of Guangzhou Municipality. We downloaded daily data of ambient temperature from China Meteorological Data Sharing Service System [22] and the data of concentration of fine particulate matter with aerodynamic diameters < 2.5 *μm* (PM_2.5_) were extracted from China’s National Urban Air Quality Real-time Publishing Platform [23].

### Data analysis

We applied Poisson regression models to the daily mortality data of various causes of death between 1 January 2018 and 20 January 2020, accounting for the effects of population size, calendar time, ambient temperature and PM_2.5_. The model was as follows:

1 $$ {Y}_t\sim Poisson\left({\mu}_t\right)\kern0.2em \log \left({\mu}_t\right)=\alpha + offset\left[\log \left({pop}_t\right)\right]+{\beta}_1{holiday}_t+{\beta}_2{year}_t+{\beta}_3\sin \left(\frac{2{\pi t}_y}{365.2425}\right)+{\beta}_4\cos \left(\frac{2{\pi t}_y}{365.2425}\right)+{\beta}_5\sin \left(\frac{4{\pi t}_y}{365.2425}\right)+{\beta}_6\cos \left(\frac{4{\pi t}_y}{365.2425}\right)+{\beta}_7{PM}_{2.5t}+{\beta}_8{Temp}_{t,l} $$

where *Y*_*t*_and *μ*_*t*_ represent observed and expected number of deaths at the day *t*. *holiday*_*t*_ is an indicator variable of holiday (1: yes; 0: no). *year*_*t*_ and *pop*_*t*_ are the year and population size at the day *t*. *t*_*y*_ indicates day of the year (1,2,3…365). A linear function was used for the 2-day moving average of current- and previous-day PM_2.5_ [24]. *Temp*_*tll*_ is a cross-basis matrix obtained by applying a distributed lag non-linear model to ambient temperature and lag. Here, we employed a natural cubic spline with four degrees of freedom (*df*s) for both of temperature and lag with a maximum lag of 21 days [25].

Next, we explored whether the mortality impacts of COVID-19 outbreak were different between in-hospital deaths and the deaths occurring outside hospitals. Additionally, to examine whether the impacts of COVID-19 outbreak differed by sociodemographic status, subgroup analyses were further performed by age group (i.e. < 25, 25–44, 45–64, 65–74, 75–84, 85+ years), sex, marital status (i.e. unmarried, married, divorced, widowed) and occupation class (i.e. gold-collar worker: manager and professional; white-collar worker: staff, civil servant, soldier; pink-collar worker: freelancer and self-employed person; blue-collar worker: worker and peasantry; others: the retired, student, the unemployed and others). We used the Model I which was expressed as equation 1 with additional inclusion of age group and sex as independent variables to evaluate the mortality impacts of the COVID-19 outbreak by age and sex. In addition, we applied the model with inclusion of the variable which indicates place of death and four sociodemographic variables to explore the difference in mortality impacts by these factors, without adjustment for the population size for subgroups because of unavailability of data which is assumed to be stable during the two-year study period in the same city.

Based on the fitted models mentioned above, the expected mortality was estimated for the period from 21 January through 30 June 2020 assuming that the effects of factors aforementioned remained the same as described in the models. Excess mortality was defined as the difference between the observed mortality and the expected mortality in the absence of COVID-19 outbreak from 21 January through 30 June 2020. We estimated the excess or deficit in deaths for each day and summed them up to obtain the excess mortality which suggested the impact of COVID-19 outbreak on the population mortality in Guangzhou. In addition, we estimated the percentage change in deaths through dividing excess deaths by expected mortality. Empirical confidence intervals at 95% (95% eCIs) were estimated using the Monte Carlo simulation for the coefficients of independent variables assuming the coefficients followed a multivariate normal distribution. Temporal trends of percentage changes in excess deaths are presented on a weekly basis. We defined 31 December 2019 as the starting day of the first week so that 21 January 2020 can be the first day of a week and regarded the week in which the first day of a month lies as the first week of the month. We used R statistical software (version 3.6.2) to perform all analyses.

## Results

**Fig. 1:**
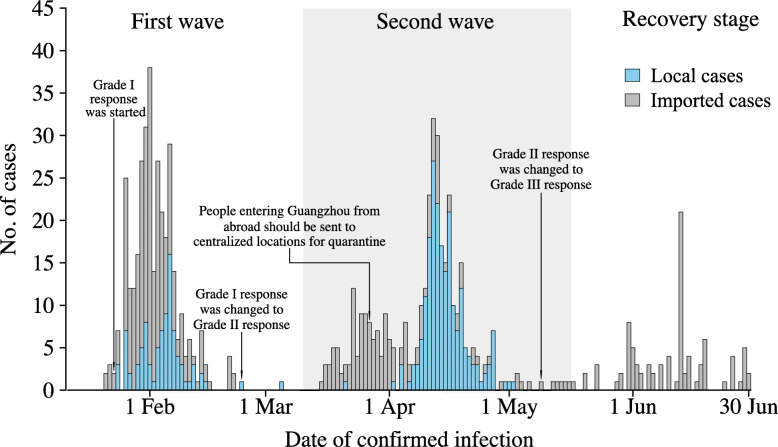
Number of cases of coronavirus disease 2019 reported in Guangzhou, China from 21 January through 30 June 2020

**Fig. 2:**
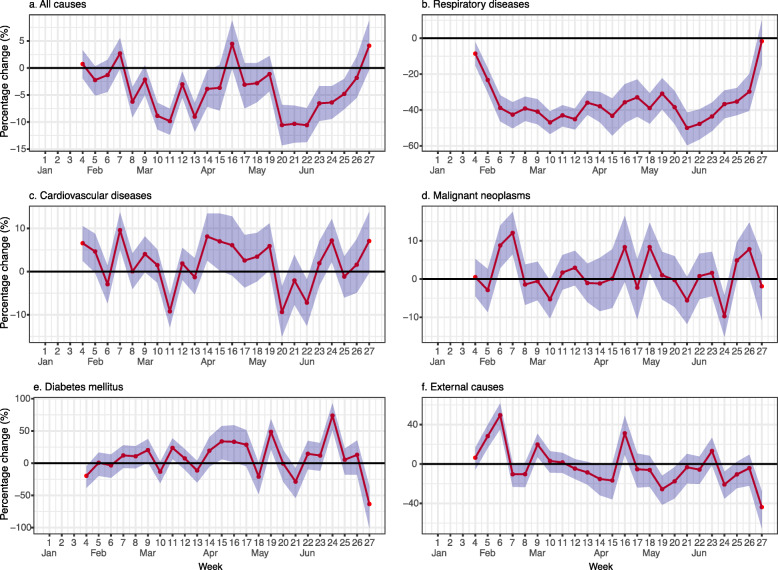
Temporal trends in percentage changes in deaths from six main causes in Guangzhou, China from 21 January through 30 June 2020. **a** all causes; **b** respiratory diseases; **c** cardiovascular diseases; **d** malignant neoplasms; **e** diabetes mellitus; **f** external causes. Red dots are point estimates of percentage changes, while grey areas are the corresponding 95% empirical confidence intervals

From 21 January through 30 June 2020, there were two waves of COVID-19 in Guangzhou. The first wave was the epidemic of domestic cases, starting on 21 January when the first COVID-19 case from Wuhan was reported, while the second wave was mainly imported cases from abroad, beginning on 11 March 2020 when the first imported case of SARS-CoV-2 infection from abroad was reported. The last local case in the second wave was reported on 2 May 2020. Afterwards, a few imported cases from abroad were reported occasionally. The recovery stage started 14 days after the last local case during the study period was reported (Fig. 1). A total of 24,340 deaths were registered in Guangzhou in the study period. Substantial temporal variations were observed in excess deaths from different causes (Fig. 2 and Additional file 1). During the first wave of COVID-19, excesses in deaths from cardiovascular diseases and malignant neoplasms occurred in February, particularly, excess was observed in hypertension mortality immediately after the start of the first wave, whereas the deficit in fatalities from respiratory diseases widened slightly (Fig. 2 and Additional file 1). After that, decreased all-cause deaths from the expected mortality were observed between late February and early April. From the 15th week through the 19th week of 2020, no significant change in mortality occurred in all-cause deaths, after which, there was a deficit in the next six-week period (Fig. 2). Increases in deaths due to hypertension and myocardial infraction were observed after the start of the second wave (Additional file 1). Notably, excess deaths due to intentional self-harm reached their highest point in the third week of April (Additional file 1). The observed respiratory deaths and the corresponding subcategories were largely lower than the expected mortality (Fig. 2 and Additional file 1).

We estimated a statistically significant deficit of 1051 (95% eCI: 580, 1558) deaths during the study period (Table 1). Remarkable decreases were observed in the numbers of deaths due to respiratory diseases (excess deaths: − 1691 [95% eCI: − 1929, − 1459]; percentage change: − 37.4%) and the subcategories, including pneumonia and influenza (− 1383 [95% eCI: − 1595, − 1196]; − 49.2%) and chronic lower respiratory disease (− 342 [95% eCI: − 474, − 223]; − 22.1%) compared with the expected mortality (Table 1). However, increases were seen in the numbers of deaths due to two subcategories of cardiovascular diseases including hypertension (113 [95% eCI: 16, 194]; 14.5%) and myocardial infarction (156 [95% eCI: 4, 278]; 8.6%). Changes in deaths due to other causes were statistically non-significant (Table 1).

**Table 1:** Estimated excess mortality and percentage changes in deaths from different causes in Guangzhou, China from 21 January through 30 June 2020

Cause of death	Excess mortality (95% eCI)	Percentage change % (95% eCI)
All causes	-1051 (−1558, − 580)	− 4.1 (− 6.1, − 2.3)
Respiratory diseases	−1691 (− 1929, − 1459)	−37.4 (− 42.6, − 32.2)
Pneumonia and influenza	−1383 (− 1595, − 1196)	−49.2 (− 56.7, − 42.6)
Chronic lower respiratory diseases	−342 (− 474, − 223)	−22.1 (− 30.6, − 14.4)
Cardiovascular diseases	197 (−156, 516)	1.9 (− 1.5, 5.0)
Hypertension	113 (16, 194)	14.5 (2.0, 24.8)
Myocardial infarction	156 (4, 278)	8.6 (0.2, 15.4)
Cerebrovascular diseases	13 (−189, 208)	0.3 (−4.5, 4.9)
Malignant neoplasms	80 (−180, 305)	1.3 (−2.8, 4.8)
Malignant neoplasm of liver and intrahepatic bile dusts	7 (− 97, 93)	0.7 (−10.1, 9.7)
Malignant neoplasm of the trachea, bronchus and lung	74 (−55, 184)	4.1 (−3.1, 10.2)
Diabetes mellitus	58 (−21, 124)	10.2 (−3.7, 22.0)
External causes	−6 (− 114, 88)	−0.5 (−9.2, 7.2)
Transport accidents	−15 (−58, 15)	−8.8 (− 33.2, 8.3)
Intentional self-harm	3 (−51, 39)	1.7 (−27.5, 21.1)

*Abbreviation*: *95% eCI* 95% Empirical confidence interval

**Fig. 3:**
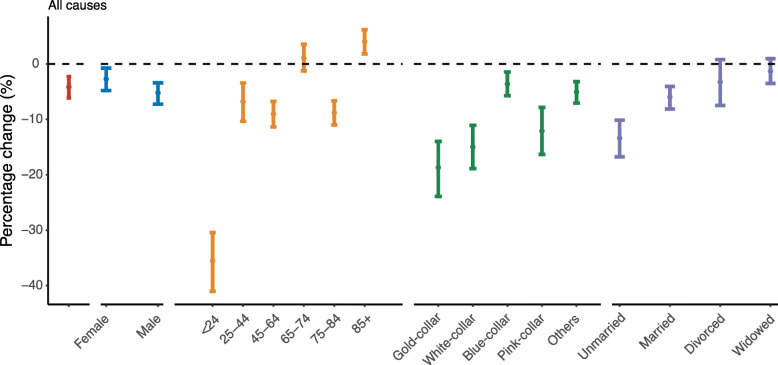
Percentage changes in all-cause deaths by sex, age, marital status and occupation class in Guangzhou, China from 21 January through 30 June 2020. Dots are point estimates of percentage changes, while lines are the corresponding 95% empirical confidence intervals

Among the six age groups, mortality deficits were seen in individuals aged < 25 (− 35.5%), 25–44, (− 6.8%), 45–64 (− 9.0%) and 75–84 years (− 8.8%), while excesses in deaths occurred in those aged 85 years or older (4.0%) (Fig. 3 and Additional file 2). Among the individuals aged 85 years or over, excesses were observed particularly in deaths due to cardiovascular diseases (11.1%) and the subcategories (hypertension: 28.8%; myocardial infarction: 21.2%; cerebrovascular diseases: 8.7%), malignant neoplasm of liver and intrahepatic bile dusts (24.6%), diabetes mellitus (37.6%) and external causes (14.5%) (Additional file 2 and Additional file 3). Deaths due to transport accidents were 24.0% below the expected mortality for residents aged < 25 years, while the differences between actual and expected mortality were non-significant for other age groups (Additional file 3). Sex difference in the percentage change in deaths was inconclusive for various causes of death, with overlapping 95% eCIs (Fig. 3, Additional file 2 and Additional file 3).

**Fig. 4:**
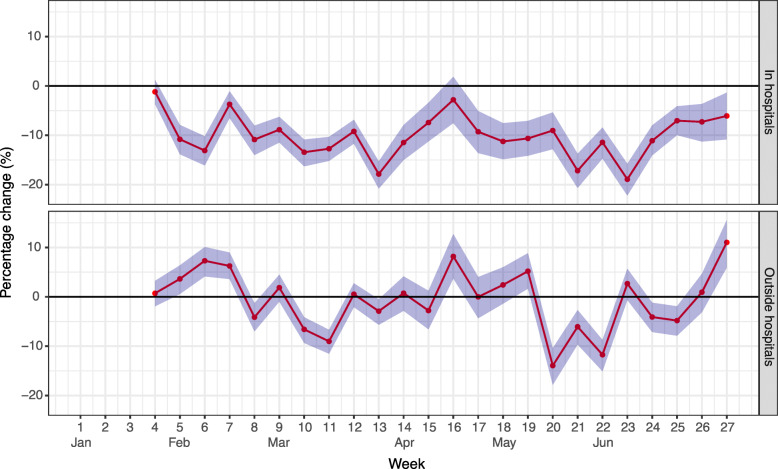
Temporal trends in percentage changes in all-cause deaths by place of death in Guangzhou, China from 21 January through 30 June 2020. Red dots are point estimates of percentage changes, while grey areas are the corresponding 95% empirical confidence intervals

It was estimated that all-cause deaths occurring in hospitals declined 10.2% from the expected mortality in the absence of COVID-19 outbreak, while the percentage change in deaths happening outside hospitals was not statistically significant (Additional file 4). An excess in deaths occurred outside hospitals in February, while a deficit was observed in deaths happening in hospitals (Fig. 4).

Percentage changes in deaths from various causes by marital status and occupation class are presented in Fig. 3, Additional file 4 and Additional file 5. For marital status, a deficit in all-cause deaths was seen in the unmarried (− 13.4%) and married (− 6.0%), while deaths in the divorced and widowed were statistically non-significant (Fig. 3 and Additional file 4). When examined by occupation class, some subgroups experienced disproportionate percentage changes in all-cause deaths. Specifically, all-cause deaths were 18.7, 15.0 and 12.1% below the expected mortality for gold-collar, white-collar and pink-collar workers over the study period, and the changes in deaths for other subgroups were smaller (Fig. 3 and Additional file 4).

## Discussion

In this study, we reported the excess mortality of the entire circle of COVID-19 outbreak from 21 January through 30 June in 2020 in Guangzhou, China. During this period, there were three stages of the COVID-19 outbreak, including the first wave, second wave and recovery stage that started on 21 January, 11 March, and 17 May, respectively. Regarding the temporal trends of mortality impacts of COVID-19, excess deaths from cardiovascular diseases and malignant neoplasms occurred in February. At the beginning of SARS-CoV-2 transmission, the virus infection can result in increased deaths due to the virus infection itself, overburdened health care systems and depleted resources. Deaths would also occur in patients who need disease interventions but are reluctant to seek professional help during the outbreak [26–28]. Excesses in deaths from hypertension and myocardial infraction seen in the second wave might be in part attributable to the delays in treatment among patients with the underlying health conditions. The deficit in deaths happening in hospitals also suggested the potential delays in treatment. Essential services are recommended to be maintained and patients with related chronic diseases should be given priority when allocating patient care resources [12]. Deficit in all-cause deaths was seen between late February and early April. Interventions of social distancing, such as the requirement of wearing a face mask outdoors and encouraging residents staying at home may contribute to the mortality deficit through reducing 1) the exposure to other viruses which are causes of infectious diseases and 2) traffic volume that deems to be related to traffic injuries [29]. Indirect impacts of the COVID-19 outbreak would in addition comprise recession and the subsequent shortage of food, drugs and other necessities, disruption of social networks and the potential mental health problem in the population [29]. In this study, we observed that the excess deaths due to intentional self-harm reached their highest point in the third week of April. The excess mortality only represented the short-term impact of COVID-19 outbreak. It is anticipated that the impact of COVID-19 on mental health can be prolonged [13]. Efforts, such as providing public psychological consultation for the people who need it, are required to mitigate the short- and long-term disease burden among resident with mental health conditions.

Overall, we observed a statistically significant deficit in mortality during the COVID-19 outbreak in Guangzhou, which was extremely distinct from other countries, such as the United States, Italy and the England and Wales where the death tolls were 18.6–55.5% above the expected mortality [10, 13, 18]. The reason is mainly due to be the differences in the severity and the stage of COVID-19 outbreak under study (most of the previous studies focused on the early or mild stages of COVID-19 outbreak) and partly due to the differences in quality of and access to health care services, the effectiveness of the measures taken to contain COVID-19 and other influential factors of mortality that were related to the containing measures. The deficit in all-cause deaths occurred during the COVID-19 outbreak in Guangzhou highlights the effectiveness of implementing sustained and rigorous suppression measures in response to the outbreak [30]. As the control of the outbreak and the availability of vaccine, the control measures have changed accordingly to minimize its impact on social daily life and economic development.

We estimated that deaths due to hypertension and myocardial infarction were 14.5 and 8.6% higher than the expected mortality. Excesses in deaths due to hypertension and myocardial infarction were also reported in England and Wales [13]. Social distancing and isolation may result in increased stress which can lead to exacerbation of hypertension and myocardial infarction and subsequently cause deaths. In addition, not receiving necessary treatment during the COVID-19 outbreak may also result in excess deaths. Patients with conditions which would be deteriorated by stress such as hypertension and myocardial infarction should be given sufficient attention when an infectious disease outbreak occurs.

Notably, we observed a significant deficit in deaths due to respiratory diseases and the subcategories including pneumonia and influenza and chronic lower respiratory diseases during the study period. The reduction in patients staying in hospitals may avoid hospital infection and related severe clinical outcomes. In addition, the implementation of social distancing measures, such as the requirement of wearing a face mask, promoting healthy hygiene practices and residents’ cooperation in Guangzhou have curbed the transmission of SARS-CoV-2 virus as well as other respiratory viruses and thereafter mitigated the associated disease burden.

There is no consensus on the sex difference in the excess mortality during the COVID-19 outbreak. Two studies reported higher excess deaths in male than in females [11, 31], while inconclusive sex difference was reported in several locations [17, 20]. Similarly, we did not identify a significant sex difference. Differential indices applied for the assessment, age structures and study periods may account for the distinct findings [13, 18, 20, 32]. Percentage change in deaths compared with the expected mortality would allow for a fair comparison between males and females. We observed a variation in the excess mortality across different age group, with the vast majority of excess deaths occurring in those aged 85 years or over. The large mortality burden in this age group may in part due to the deterioration of multiple comorbidities triggered by SARS-CoV-2 virus infection, the lack of physical activity or the delays in necessary treatment, while reduced exposure to viruses that lead to infectious diseases may contribute to the deficit in deaths for other age groups, including those aged 0–64 and 75–84 years.

We found a deficit in all-cause deaths in the unmarried and it was larger than those in others. One possible reason for this finding is that interventions of social distancing change the behavior pattern for a larger proportion of the unmarried compared with other subgroups, as the unmarried are on average younger than others. For instance, deaths due to transport accidents reduced − 21.9% from the expected mortality among the unmarried (Additional file 5). Further, our study offered interesting clues on differentials in occupation class. We found all-cause deaths were more than 10% lower for gold-collar, white-collar and pink-collar workers. Differences in age structure, comorbidity and the stress from work for different subgroups may explain the disproportionate mortality burden by occupation class.

Our study is subject to several limitations. First, very few deaths may be underreported due to the registration delay. However, each death must be registered 2 weeks after the certification of death in Guangzhou. Next, we did not consider other influential factors of death, such as influenza and respiratory syncytial virus, since the data were not available. Further, we only examined the temporal pattern in the excess mortality during the COVID-19 outbreak from 21 January through 30 June 2020. However, the impact can be prolonged especially for the individuals with chronic diseases and psychological illnesses [13]. This issue can be further addressed with additional data and information for a longer period of time. Last, the current study did not conclusively provide the mechanisms for the occurrence of excess deaths but may afford us some timely indications. Lastly, the death register system only covers permanent residents. Excess mortality was not evaluated for temporary population.

## Conclusions

In conclusion, excess deaths fluctuate during the COVID-19 outbreak in Guangzhou, China. Overall, a deficit in deaths occurred during the COVID-19 outbreak in Guangzhou. It is recommended to maintain essential services especially for the elderly and patients with comorbid conditions. Our findings have important implications on how to allocate resources to combat COVID-19 and alleviate its adverse effect in different stages of the outbreak.

## Supplementary Information

**Additional file 1: Figure S1.** Temporal trends in percentage changes in deaths from nine subcategories of causes in Guangzhou, China from 21 January through 30 June 2020. (a) pneumonia and influenza; (b) chronic lower respiratory diseases; (c) hypertension; (d) myocardial infarction; (e) cerebrovascular diseases; (f) malignant neoplasm of liver and intrahepatic bile dusts; (g) malignant neoplasm of the trachea, bronchus and lung; (h) transport accidents; (i) intentional self-harm. Red dots are point estimates of percentage changes, while grey areas are the corresponding 95% empirical confidence intervals.

**Additional file 2: Table S1.** Percentage changes in deaths from six main causes by sex and age in Guangzhou, China from 21 January through 30 June 2020.

**Additional file 3: Table S2.** Percentage changes in deaths from nine subcategories of causes by sex and age in Guangzhou, China from 21 January through 30 June 2020.

**Additional file 4: Table S3.** Percentage changes in deaths from six main causes by place of death, marital status and occupation class in Guangzhou, China from 21 January through 30 June 2020.

**Additional file 5: Table S4.** Percentage changes in deaths from nine subcategories of causes by place of death, marital status and occupation class in Guangzhou, China from 21 January through 30 June 2020.

## Data Availability

Data that support the findings of current study are available from Guangzhou CDC, but restrictions apply to the availability of these data. Permission can be requested by contacting PZQ.

## Abbreviations

COVID-19: Coronavirus disease 2019
SARS-CoV-2: Severe acute respiratory syndrome coronavirus 2
ICD-10: International statistical classification of diseases and related health problems 10th revision
CDC: Center for disease control prevention
PM_2.5_: Fine particulate matter with aerodynamic diameters < 2.5 *μm*
