# Supplementary material for: Temporal dynamic in the impact of COVID− 19 outbreak on cause-specific mortality in Guangzhou, China
Source: BMC Public Health. 2021 May 8;21:883. doi: 10.1186/s12889-021-10771-3 (PMC8105693; doi:10.1186/s12889-021-10771-3)
Supplement: Supplementary file 1 — Additional file 1: Figure S1. Temporal trends in percentage changes in deaths from nine subcategories of causes in Guangzhou, China from 21 January through 30 June 2020. (a) pneumonia and influenza; (b) chronic lower respiratory diseases; (c) hypertension; (d) myocardial infarction; (e) cerebrovascular diseases; (f) malignant neoplasm of liver and intrahepatic bile dusts; (g) malignant neoplasm of the trachea, bronchus and lung; (h) transport accidents; (i) intentional self-harm. Red dots are point estimates of percentage changes, while grey areas are the corresponding 95% empirical confidence intervals. [file 12889_2021_10771_MOESM1_ESM.pdf]

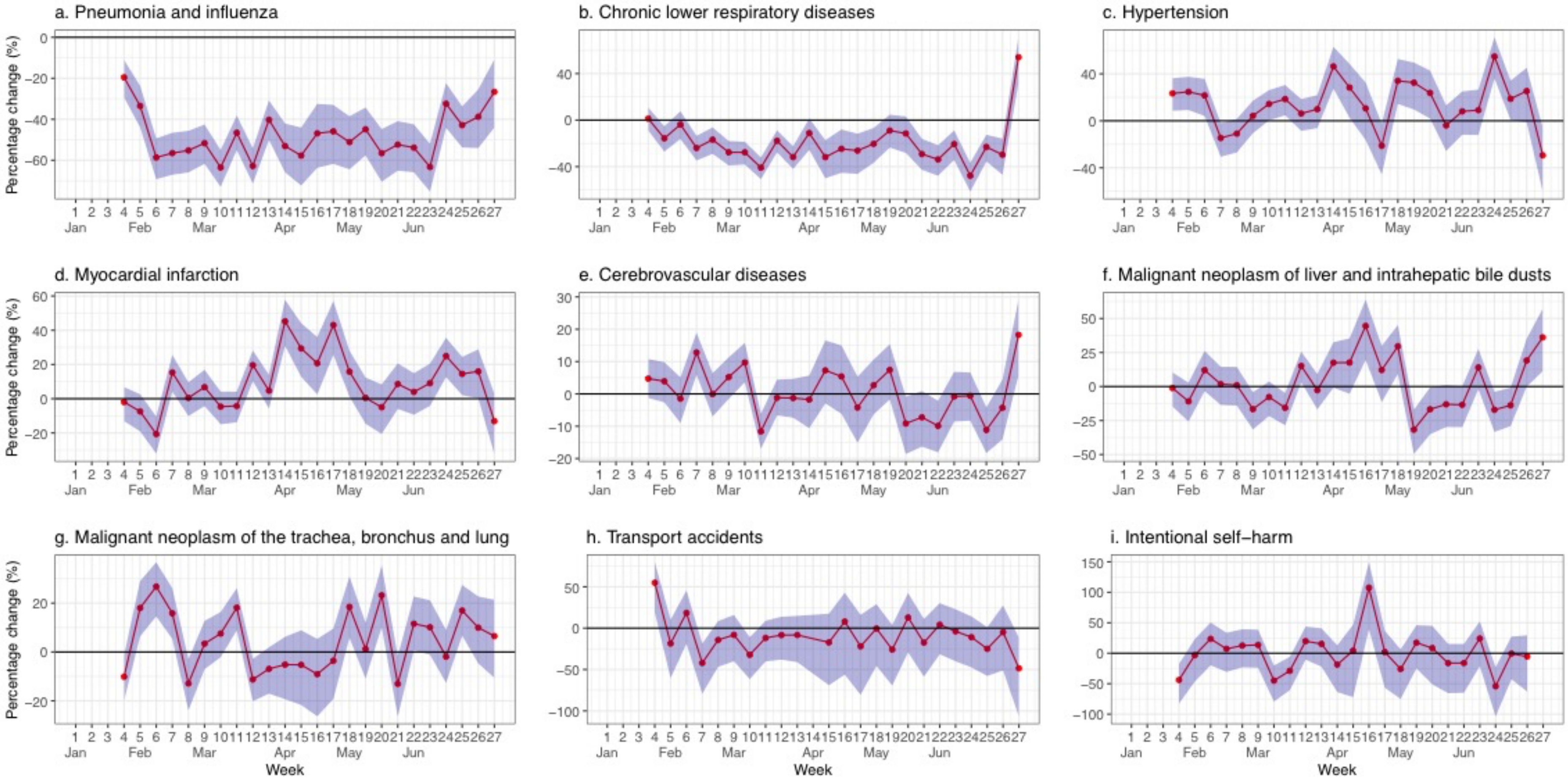

Additional file 1: Figure S1. Temporal trends in percentage changes in deaths from nine subcategories of causes in Guangzhou, China from 21 January through 30 June 2020. (a) pneumonia and influenza; (b) chronic lower respiratory

diseases; (c) hypertension; (d) myocardial infarction; (e) cerebrovascular diseases; (f) malignant neoplasm of liver and intrahepatic bile dusts; (g) malignant neoplasm of the trachea, bronchus and lung; (h) transport accidents; (i) intentional self-harm. Red dots are point estimates of percentage changes, while grey areas are the corresponding 95% empirical confidence intervals.
