# Supplementary material for: Temporal dynamic in the impact of COVID− 19 outbreak on cause-specific mortality in Guangzhou, China
Source: BMC Public Health. 2021 May 8;21:883. doi: 10.1186/s12889-021-10771-3 (PMC8105693; doi:10.1186/s12889-021-10771-3)
Supplement: Supplementary file 2 — Additional file 2: Table S1. Percentage changes in deaths from six main causes by sex and age in Guangzhou, China from 21 January through 30 June 2020. [file 12889_2021_10771_MOESM2_ESM.pdf]

Additional file 2: Table S1. Percentage changes in deaths from six main causes by sex and age in Guangzhou, China from 21 January through 30 June 2020.

Category	Percentage change % (95% eCI)					
	All causes	Respiratory diseases	Cardiovascular diseases	Malignant neoplasms	Diabetes mellitus	External causes
Sex						
Male	-5.2 (-7.3, -3.4)	-37.5 (-42.9, -32.8)	1.6 (-1.9, 4.6)	1.4 (-2.4, 5.1)	13.5 (-1.4, 25.6)	-3.3 (-13.4, 5.1)
Female	-2.7 (-4.8, -0.7)	-37.1 (-42.9, -32.1)	2.3 (-0.8, 5.4)	1.0 (-3.0, 4.6)	6.7 (-9.2, 19.0)	3.1 (-7.4, 11.8)
Age group, years						
<25	-35.5 (-41.0, -30.4)	-65.1 (-84.3, -49.4)	-24.4 (-46.5, -5.3)	-13.6 (-29.4, -0.4)	120.4 (-31.6, 182.8)	-14.0 (-28.4, -1.9)
25-44	-6.8 (-10.4, -3.4)	-29.6 (-49.1, -13.4)	12.9 (4.6, 20.5)	1.7 (-4.5, 7.5)	2.3 (-33.2, 26.6)	-23.0 (-35.1, -12.3)
45-64	-9.0 (-11.4, -6.8)	-35.3 (-42.3, -28.2)	-9.6 (-13.5, -6.0)	0.6 (-3.7, 4.6)	4.5 (-13.1, 16.9)	-13.0 (-23.8, -4.0)
65-74	1.1 (-1.3, 3.5)	-33.3 (-40.1, -27.1)	5.3 (1.6, 9.0)	8.6 (4.2, 12.7)	6.7 (-10.5, 19.4)	6.5 (-5.7, 17.1)
75-84	-8.8 (-11.0, -6.6)	-42.0 (-47.4, -36.7)	-3.5 (-7.0, -0.2)	-4.7 (-9.1, -0.5)	4.3 (-12.0, 16.5)	11.4 (0.4, 21.0)
85+	4.0 (1.8, 6.2)	-34.3 (-39.9, -29.1)	11.1 (7.8, 14.2)	0.2 (-4.9, 5.2)	37.6 (19.7, 52.0)	14.5 (3.5, 23.5)

Abbreviation: 95% eCI, 95% empirical confidence interval.
