# Supplementary material for: Temporal dynamic in the impact of COVID− 19 outbreak on cause-specific mortality in Guangzhou, China
Source: BMC Public Health. 2021 May 8;21:883. doi: 10.1186/s12889-021-10771-3 (PMC8105693; doi:10.1186/s12889-021-10771-3)
Supplement: Supplementary file 3 — Additional file 3: Table S2. Percentage changes in deaths from nine subcategories of causes by sex and age in Guangzhou, China from 21 January through 30 June 2020. [file 12889_2021_10771_MOESM3_ESM.pdf]

Additional file 3: Table S2. Percentage changes in deaths from nine subcategories of causes by sex and age in Guangzhou, China from 21 January through 30 June 2020.

Category	Percentage change % (95% eCI)								
	**Pneumonia and influenza**	**Chronic lower respiratory diseases**	**Hypertension**	**Myocardial infarction**	**Cerebrovascular diseases**	**Malignant neoplasm of liver and intrahepatic bile dusts**	**Malignant neoplasm of the trachea, bronchus and lung**	**Transport accidents**	**Intentional self-harm**
Sex									
Male	-50.7 (-58.2, -44.7)	-21.4 (-30.3, -13.9)	17.2 (4.5, 27.5)	7.4 (-0.5, 14.5)	-3.2 (-8.5, 1.3)	0.0 (-11.2, 9.1)	3.4 (-4.2, 10.5)	-11.6 (-36.6, 6.1)	8.1 (-21.4, 27.5)
Female	-47.3 (-55.2, -40.8)	-23.9 (-33.4, -15.4)	12.3 (-0.6, 22.3)	10.4 (1.8, 17.6)	4.3 (-0.9, 9.2)	3.3 (-8.6, 13.5)	5.6 (-2.8, 13.1)	-1.7 (-29.8, 16.8)	-7.7 (-40.5, 12.0)
Age group, years									
<25	-71.7 (-92.8, -53.7)	-100.0 (-268.9, -36.5)		-41.3 (-145.3, 9.4)	-22.1 (-71.6, 12.8)	-41.6 (-153.5, 7.2)	-100.0 (-315.2, -31.9)	-24.0 (-62.2, -2.6)	3.3 (-37.9, 27.1)
25-44	-30.8 (-54.6, -11.1)	-28.0 (-90.3, 10.0)	-2.0 (-52.4, 27.5)	23.5 (5.4, 37.6)	13.2 (-0.6, 25.4)	-2.2 (-16.3, 10.5)	8.0 (-11.7, 23.5)	-9.5 (-39.2, 9.9)	-11.0 (-43.3, 10.0)
45-64	-49.6 (-60.2, -40.1)	-13.6 (-28.8, -2.4)	-15.7 (-31.3, -2.5)	4.2 (-4.9, 12.5)	-13.6 (-19.8, -7.5)	-1.4 (-12.9, 8.1)	0.7 (-7.6, 7.7)	-16.3 (-41.7, 2.0)	-5.1 (-37.1, 14.0)
65-74	-46.8 (-56.4, -38.8)	-14.4 (-25.2, -4.7)	13.6 (-2.0, 26.6)	4.2 (-5.0, 12.3)	5.0 (-0.9, 10.7)	1.4 (-10.4, 11.4)	8.8 (0.5, 16.0)	-1.8 (-30.0, 17.7)	37.3 (0.5, 61.5)

75-84	-50.1 (-58.8, -43.2)	-31.6 (-40.7, -23.5)	8.8 (-3.6, 19.7)	2.7 (-6.3, 10.2)	-3.4 (-9.0, 1.8)	-0.6 (-13.8, 9.9)	1.3 (-6.7, 8.5)	13.1 (-20.4, 33.8)	7.9 (-30.1, 33.6)
85+	-48.8 (-56.8, -41.8)	-15.7 (-25.8, -7.6)	28.8 (16.5, 39.1)	21.2 (12.2, 29.2)	8.7 (3.1, 13.4)	24.6 (8.3, 37.9)	9.1 (-2.3, 17.8)	17.2 (-43.4, 51.2)	6.5 (-54.5, 40.7)

Abbreviation: 95% eCI, 95% empirical confidence interval.
