# Supplementary material for: Temporal dynamic in the impact of COVID− 19 outbreak on cause-specific mortality in Guangzhou, China
Source: BMC Public Health. 2021 May 8;21:883. doi: 10.1186/s12889-021-10771-3 (PMC8105693; doi:10.1186/s12889-021-10771-3)
Supplement: Supplementary file 4 — Additional file 4: Table S3. Percentage changes in deaths from six main causes by place of death, marital status and occupation class in Guangzhou, China from 21 January through 30 June 2020. [file 12889_2021_10771_MOESM4_ESM.pdf]

Additional file 4: Table S3. Percentage changes in deaths from six main causes by place of death, marital status and occupation class in Guangzhou, China from 21 January through 30 June 2020.

**Category**	**Percentage change % (95% eCI)**					
	**All causes**	**Respiratory diseases**	**Cardiovascular diseases**	**Malignant neoplasms**	**Diabetes mellitus**	**External causes**
Place of death						
In hospitals	-10.2 (-12.3, -8.0)	-38.1 (-43.8, -33.0)	-3.8 (-7.4, -0.5)	-5.4 (-9.5, -1.8)	6.7 (-10.7, 18.5)	0.0 (-10.7, 9.2)
Outside hospitals	-0.8 (-2.8, 1.3)	-38.1 (-43.7, -33.0)	3.6 (0.4, 6.9)	7.6 (3.6, 11.2)	10.3 (-5.4, 21.7)	-2.3 (-12.8, 5.7)
Marital status						
Unmarried	-13.4 (-16.8, -10.1)	-42.6 (-52.5, -34.0)	13.5 (7.1, 18.9)	-1.3 (-8.9, 5.3)	16.5 (-13.8, 38.1)	-10.6 (-23.9, -1.4)
Married	-6.0 (-8.1, -4.1)	-37.2 (-42.7, -32.2)	-1.7 (-5.1, 1.5)	-0.2 (-4.0, 3.4)	6.4 (-8.2, 18.4)	-3.4 (-13.0, 4.3)
Divorced	-3.3 (-7.5, 0.8)	-42.2 (-55.1, -30.4)	4.2 (-3.2, 10.7)	10.8 (2.8, 17.7)	0.2 (-31.7, 21.3)	-15.9 (-34.0, -1.7)
Widowed	-1.3 (-3.5, 0.9)	-38.9 (-44.4, -33.8)	4.0 (0.3, 7.2)	-0.1 (-4.7, 4.6)	15.5 (-0.4, 27.7)	17.3 (6.3, 25.8)
Occupation class						
Gold-collar	-18.7 (-23.9, -14.0)	-44.0 (-60.4, -29.7)	-15.5 (-25.9, -7.1)	-19.0 (-27.8, -11.1)	-15.0 (-52.3, 10.2)	14.1 (-10.0, 31.9)
White-collar	-15.0 (-18.9, -11.1)	-45.3 (-58.2, -33.5)	-10.3 (-18.5, -3.0)	-8.1 (-14.9, -1.9)	28.2 (-11.4, 52.8)	-8.0 (-27.1, 6.2)
Pink-collar	-12.1 (-16.3, -7.8)	-38.5 (-55.3, -24.5)	3.8 (-4.5, 10.7)	-12.2 (-19.2, -5.6)	-2.3 (-34.5, 23.5)	-14.3 (-31.3, -0.2)
Blue-collar	-3.6 (-5.7, -1.4)	-37.2 (-43.5, -32.0)	-3.1 (-6.5, -0.2)	3.8 (-0.8, 7.7)	2.6 (-13.9, 15.8)	2.2 (-7.8, 10.7)
Others	-5.1 (-7.1, -3.2)	-38.1 (-43.8, -33.3)	3.8 (0.7, 6.8)	0.3 (-3.8, 3.9)	13.3 (-3.2, 24.7)	-3.7 (-13.3, 4.6)

Abbreviation: 95% eCI, 95% empirical confidence interval.
