# Supplementary material for: Temporal dynamic in the impact of COVID− 19 outbreak on cause-specific mortality in Guangzhou, China
Source: BMC Public Health. 2021 May 8;21:883. doi: 10.1186/s12889-021-10771-3 (PMC8105693; doi:10.1186/s12889-021-10771-3)
Supplement: Supplementary file 5 — Additional file 5: Table S4. Percentage changes in deaths from nine subcategories of causes by place of death, marital status and occupation class in Guangzhou, China from 21 January through 30 June 2020. [file 12889_2021_10771_MOESM5_ESM.pdf]

Additional file 5: Table S4. Percentage changes in deaths from nine subcategories of causes by place of death, marital status and occupation class in Guangzhou, China from 21 January through 30 June 2020.

**Category**	**Percentage change % (95% eCI)**								
	**Pneumonia and influenza**	**Chronic lower respiratory diseases**	**Hypertension**	**Myocardial infarction**	**Cerebrovascular diseases**	**Malignant neoplasm of liver and intrahepatic bile dusts**	**Malignant neoplasm of the trachea, bronchus and lung**	**Transport accidents**	**Intentional self-harm**
Place of death									
In hospitals	-47.4 (-55.0, -41.3)	-30.6 (-39.7, -22.2)	14.2 (-0.8, 26.5)	-2.3 (-10.0, 5.0)	-2.3 (-7.8, 2.4)	-0.2 (-11.8, 8.4)	-4.3 (-12.4, 2.2)	2.4 (-24.3, 21.6)	11.0 (-22.5, 31.2)
Outside hospitals	-57.8 (-66.0, -51.2)	-17.4 (-26.1, -10.1)	12.7 (-1.5, 23.3)	12.4 (4.9, 19.2)	0.1 (-5.1, 4.5)	-0.7 (-11.6, 8.3)	12.3 (4.0, 19.3)	-19.1 (-46.1, -2.0)	-1.7 (-31.9, 15.2)
Marital status									
Unmarried	-55.8 (-68.2, -44.7)	-20.1 (-38.0, -5.3)	-7.0 (-33.2, 15.2)	30.7 (12.9, 44.8)	9.8 (-0.8, 18.8)	-2.2 (-23.4, 14.6)	32.9 (15.6, 47.6)	-21.9 (-51.2, -1.9)	2.9 (-31.4, 24.4)
Married	-48.0 (-55.7, -41.3)	-23.0 (-31.5, -15.6)	11.6 (-1.4, 21.9)	6.9 (-1.5, 13.9)	-3.6 (-9.0, 1.2)	-2.9 (-13.8, 6.0)	1.8 (-5.9, 8.0)	-9.6 (-36.2, 6.9)	5.6 (-24.5, 24.4)
Divorced	-55.8 (-73.7, -41.2)	-16.4 (-42.7, 2.5)	-6.6 (-34.2, 14.2)	29.2 (11.9, 44.5)	-2.6 (-14.3, 8.1)	-8.3 (-29.2, 8.4)	16.1 (-1.6, 29.0)	-66.9 (-119.3, -35.9)	-33.6 (-78.5, -8.1)
Widowed	-51.5 (-59.4, -44.1)	-23.5 (-33.3, -15.4)	17.0 (3.2, 27.3)	6.1 (-2.4, 13.6)	3.7 (-1.3, 8.7)	19.0 (3.7, 30.2)	3.6 (-5.3, 11.7)	62.6 (21.9, 89.0)	19.7 (-27.3, 46.5)

Occupation class									
Gold-collar	-58.2 (-78.5, -40.8)	-19.4 (-51.2, 6.4)	71.2 (26.8, 99.3)	-51.4 (-72.5, -36.3)	6.4 (-8.9, 19.8)	-23.0 (-44.9, -6.2)	-1.3 (-19.3, 14.6)	-24.5 (-84.8, 10.3)	30.4 (-28.4, 67.8)
White-collar	-58.0 (-78.8, -41.9)	-24.0 (-56.1, -3.2)	-35.1 (-72.4, -7.7)	-13.1 (-31.5, 1.0)	-4.1 (-17.2, 7.8)	-36.5 (-52.8, -23.1)	28.9 (13.2, 42.5)	7.7 (-39.4, 36.4)	-10.7 (-58.8, 17.1)
Pink-collar	-37.4 (-62.2, -16.5)	-41.5 (-68.0, -21.2)	19.8 (-20.5, 46.3)	35.4 (17.8, 50.3)	-12.2 (-26.3, -1.4)	-10.4 (-27.3, 3.1)	-24.2 (-39.6, -11.6)	-38.7 (-73.9, -14.8)	41.2 (-7.7, 69.4)
Blue-collar	-46.7 (-55.7, -38.1)	-20.8 (-29.7, -13.1)	7.2 (-6.1, 17.1)	-3.6 (-11.8, 4.0)	1.1 (-4.2, 5.8)	-1.8 (-12.2, 7.4)	7.7 (-0.7, 14.7)	-1.7 (-25.5, 16.4)	18.1 (-17.9, 39.1)
Others	-50.2 (-57.7, -43.7)	-24.1 (-32.8, -16.7)	16.5 (3.5, 26.5)	16.2 (8.6, 23.6)	-2.0 (-7.2, 2.4)	7.0 (-3.4, 15.9)	0.6 (-7.3, 7.4)	-16.6 (-43.5, 2.4)	-9.3 (-38.9, 10.5)

Abbreviation: 95% eCI, 95% empirical confidence interval.
